# Factors that influence the implementation of e-health: a systematic review of systematic reviews (an update)

**DOI:** 10.1186/s13012-016-0510-7

**Published:** 2016-10-26

**Authors:** Jamie Ross, Fiona Stevenson, Rosa Lau, Elizabeth Murray

**Affiliations:** e-Health Unit, Research Department of Primary Care and Population Health, University College London, Upper 3rd Floor, Royal Free Campus, Rowland Hill Street, London, NW3 2PF UK

**Keywords:** Implementation, e-Health, Systematic review, Update, Synthesis

## Abstract

**Background:**

There is a significant potential for e-health to deliver cost-effective, quality health care, and spending on e-health systems by governments and healthcare systems is increasing worldwide. However, there remains a tension between the use of e-health in this way and implementation. Furthermore, the large body of reviews in the e-health implementation field, often based on one particular technology, setting or health condition make it difficult to access a comprehensive and comprehensible summary of available evidence to help plan and undertake implementation. This review provides an update and re-analysis of a systematic review of the e-health implementation literature culminating in a set of accessible and usable recommendations for anyone involved or interested in the implementation of e-health.

**Methods:**

MEDLINE, EMBASE, CINAHL, PsycINFO and The Cochrane Library were searched for studies published between 2009 and 2014. Studies were included if they were systematic reviews of the implementation of e-health. Data from included studies were synthesised using the principles of meta-ethnography, and categorisation of the data was informed by the Consolidated Framework for Implementation Research (CFIR).

**Results:**

Forty-four reviews mainly from North America and Europe were included. A range of e-health technologies including electronic medical records and clinical decision support systems were represented. Healthcare settings included primary care, secondary care and home care. Factors important for implementation were identified at the levels of the following: the individual e-health technology, the outer setting, the inner setting and the individual health professionals as well as the process of implementation.

**Conclusion:**

This systematic review of reviews provides a synthesis of the literature that both acknowledges the multi-level complexity of e-health implementation and provides an accessible and useful guide for those planning implementation. New interpretations of a large amount of data across e-health systems and healthcare settings have been generated and synthesised into a set of useable recommendations for practice. This review provides a further empirical test of the CFIR and identifies areas where additional research is necessary.

**Trial registration:**

PROSPERO, CRD42015017661

**Electronic supplementary material:**

The online version of this article (doi:10.1186/s13012-016-0510-7) contains supplementary material, which is available to authorized users.

## Background

Technology is used extensively to provide and deliver health care worldwide [1, 2]. e-Health (the application of information, computer or communication technology to some aspects of health or health care) is viewed as essential for solving problems facing healthcare systems of increasing demand, due to an ageing population and improved treatments, and limited resources [3]. However, although there is widespread agreement about the importance and potential benefits of e-health, realisation of these benefits has often been slower than anticipated, often because of difficulties with implementation [4]. For example, in the UK, the National Health Service (NHS) Five Year Forward View [5] states the need to make better use of available health technologies and acknowledges that the NHS has previously failed to make best use of these because of difficulties in understanding how best to adopt and implement them. High-profile implementation failures continue to be reported, such as the failure of implementation of an e-health system in a major UK teaching hospital, leading to reduced performance, demoralised staff, costs of £200 million and the trust being put into special measures [6]. This highlights the strong need for those undertaking the implementation of e-health to understand factors that influence implementation and be well equipped to devise strategies and interventions to improve the widespread effective use of e-health and address blockages to implementation.

One problem with the current e-health implementation literature is that it is fragmented across multiple subspecialty areas [7]. With a plethora of reviews on the implementation of different e-health technologies available, it may be difficult for clinicians, managers or policymakers to locate and apply an appropriate body of evidence for their specific circumstances.

The aim of this systematic review of reviews was to provide a synthesis of the implementation of e-heath literature that both acknowledges the multi-level complexity of implementation and also provides a framework for thinking about implementation in a way that is accessible and useful for those planning implementation such as health service managers, healthcare professionals and researchers. Specific objectives were to (i) identify published reviews pertaining to implementation of e-health systems; (ii) summarise the data contained in these reviews; (iii) synthesise these data according to the Consolidated Framework for Implementation Research (CFIR) [8]; and (iv) provide recommendations for future implementations of e-health systems. The CFIR provides a systematic way of identifying the factors that are important for implementation, and its use also allows identification of areas where there is insufficient evidence and further research is required.

## Methods/design

The protocol for this systematic review has been published [9] and registered with the Prospective Register of Systematic Reviews (registration number CRD42015017661).

A systematic review of reviews by Mair et al. [7] synthesised the literature on the implementation of e-health interventions in healthcare settings published up until 2009. As the use of e-health is rapidly growing and changing, and the nature of healthcare systems are continually shifting, an update of this review was deemed timely. A systematic review of reviews was deemed to be the most appropriate method, as opposed to a systematic review of the primary literature, as the huge number of primary studies in the area would make synthesis potentially unworkable and very time consuming. A systematic review of reviews provides a summary of evidence from a variety of different levels, including the combination of different interventions, different populations and different settings [10] in a coherent and economical way [7]. Separate reviews are brought together, compared and contrasted, which allows for new insights to be generated across the literature and synthesised into a simple overview of a large body of work.

This update largely replicated the methods for identifying and selecting studies described in the original review [7] but, as detailed, differs in the methods of data analysis. For reader clarity, henceforth, the following terms shall be used to describe the reviews referred to.

- Review—the current systematic review of review
- Original review—the systematic review of reviews conducted by Mair et al. [7]
- Studies/papers—the systematic reviews identified and synthesised in this review of reviews

## Reporting

This systematic review is reported following the ENTREQ statement guidelines to enhance transparency in reporting qualitative evidence synthesis [11].

## Inclusion and exclusion criteria

The eligibility criteria for study inclusion (replicated from the Mair review) were developed using the acronym PICOS (see Table 1).

**Table 1:** Eligibility criteria for study inclusion

Population	Healthcare settings (including but not limited to primary, intermediate, secondary, home care).
	All healthcare settings were considered.
	Not limited by: clinical area, health concern; the type of patient receiving the e-health technology; the type of health professional delivering care or country.
Intervention	e-Health technologies (including management systems, such as electronic health records that allow the acquisition, transmission and storage of patient data; computerised decision support systems including diagnostic support, alerts and reminder systems; communication systems such as telecommunication that act as an intermediary between users; and information resources such as the Internet)
Comparator	This review was not limited to comparator studies.
Outcomes	Qualitative data on factors that inhibit or promote implementation of e-health.
Study type	Papers were included if they were as follows:
	• Systematic reviews: where relevant literature had been identified by means of structured search of bibliographic and other databases, where transparent methodological criteria were used to exclude papers that did not meet an explicit methodological benchmark, and which presented rigorous conclusions about outcomes.
	• Narrative reviews: where relevant literature had been purposively sampled from a field of research; where theoretical or topical criteria were used to include papers on the grounds of type, relevance and perceived significance; with the aim of summarising, discussing and critiquing conclusions.
	• Qualitative meta-syntheses or meta-ethnographies, where relevant literature was identified by means of a structured search of bibliographic and other databases, where transparent methods had been used to draw together theoretical products, with the aim of elaborating and extending theory.
	And were excluded if they were as follows:
	• Secondary analyses (including qualitative meta-syntheses or meta-ethnographies) of existing data-sets for the purposes of presenting cumulative outcomes from personal research programmes.
	• Secondary analyses (including qualitative meta-syntheses or meta-ethnographies) of existing data-sets for the purposes of presenting integrative outcomes from different research programmes.
	• Discussions of literature included in contributions to theory building or critique.
	• Summaries of literature for the purposes of information or commentary.
	• Editorial discussions that argue the case for a field of research or a course of action.
	Where an abstract stated it was a review, but there was no supporting evidence in the main paper, such as details of databases searched or criteria for selection of papers (either on methodological or theoretical grounds), the paper was excluded.

## Search strategy for identification of studies

Comprehensive electronic searches of MEDLINE, EMBASE, CINAHL, PsycINFO and The Cochrane Library were conducted.

The search strategy, which was replicated from the original review, was based on the following two concepts: e-health and implementation. The search strategy included a combination of Medical Subject Headings and free-text words. The MEDLINE (Ovid) search strategy that was used to identify papers is presented in Additional file 1. There was no limitation of language. Citation searches were carried out in ISI Web of Science in September 2015 and results were limited, in line with the search strategy, to studies published up until 1 January 2014. Reference lists of all included studies were also screened for additional literature.

The original review [7] was based on 37 papers published between 1995 and 31 July 2009. The search strategy used in the original review was replicated to identify additional literature published from 1 August 2009 until 1 January 2014. The 37 papers identified by the original review were also screened for inclusion in the current review; hence, this review includes papers identified through systematic searches of the literature published between 1995 and 2014.

## Selection of studies

**Fig. 1:**
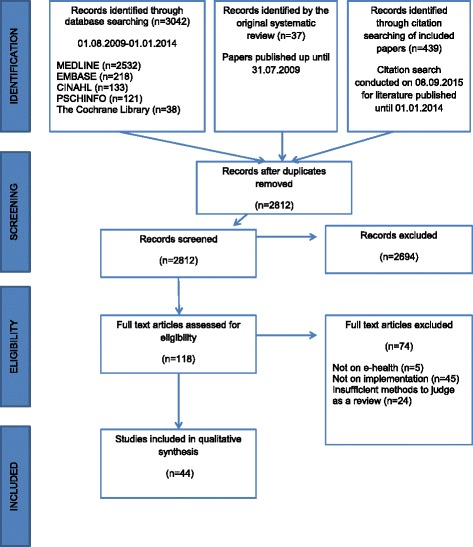
PRISMA flow diagram of study selection

Search results were imported into the EndNote reference management software, and duplicates were removed automatically and double checked manually. Titles and abstracts of all identified records were independently assessed by JR and RL. Full-text papers of references that were deemed potentially eligible were obtained and assessed for eligibility against the pre-specified selection criteria. Any discrepancies between reviewers were resolved through discussion. Reasons for exclusion at this stage were recorded and are detailed in the Preferred Reporting Items for Systematic Reviews and Meta-Analyses (PRISMA) diagram [12] (see Fig. 1).

## Study quality assessment

Data on the methodological quality of the included reviews was extracted based on ENTREQ statement guidelines [11] and was used to describe the quality of the included reviews. Because the aim was to describe and synthesise a body of qualitative literature and not determine an effect size, studies were not excluded based on this assessment.

## Data extraction

An excel spreadsheet was created for the purposes of data extraction which contained a row for each included review and columns to describe the studies and classify the extracted data related to the implementation of e-health. Data were extracted from the included studies by JR, and data extraction and coding was checked by EM.

## Data synthesis

The original review had used a thematic approach to analysing and synthesising the data, together with an analysis based on normalization process theory, which focusses on the work of implementation. In view of the large amount of new data, and the subsequent development of the CFIR [10], which pays more detailed attention to aspects such as legislative or financial frameworks, we decided to update the analytic approach to use an approach which drew on the principles of meta-ethnography for data synthesis, with the CFIR as an organising framework. The use of a framework like the CFIR aids the transferability and comparability of findings from this review to other implementation studies and allows those undertaking implementation to access the parts of this review that are of most interest to them. The CFIR [8], consolidates implementation factors from a broad array of implementation theories and is composed of five major constructs made up of components that influence the implementation of innovations into practice (see Table 2).

**Table 2:** Summary of findings of factors important for the implementation of e-health

CFIR construct	CFIR component	CFIR sub-component	Sources	e-Health domain				
				MS	CS	CD	IS	RS
Innovation characteristics	Innovation source		[16, 18]	x				
	Evidence strength and quality		[17, 18, 20, 22, 25, 53, 57]	x	x			x
	Relative advantage		[17, 25, 31, 34, 38, 43, 49, 51, 57]		x	x	x	x
	Adaptability		[16, 18, 21, 22, 24, 25, 27, 28, 34, 35, 38, 39, 41, 48–50, 52, 53, 58]	x	x	x	x	x
	Trialability		[41, 51]	x	x			
	Complexity		[18, 27, 28, 34, 38, 40, 41, 43, 45, 49, 52, 53, 55]	x		x	x	x
	Design quality and packaging		[18, 36, 43, 58]	x		x		x
	Cost		[15–20, 22–25, 27, 28, 30–33, 36–39, 41, 43, 47, 49, 51–54, 57]	x	x	x	x	x
Outer setting	Patient needs and resources		[31, 33, 38, 41, 51]	x	x	x		
	Cosmopolitanism		[22, 51]		x			
	Peer pressure		No data					
	External policy and incentives		[16–20, 24, 28, 30, 31, 33, 36, 37, 39, 48, 49, 51]	x	x	x		x
Inner setting	Structural characteristics		[18, 27, 37, 44, 49, 52, 57, 58]	x	x			x
	Networks and communications		[21, 22, 33, 37, 39, 49, 50, 58]	x	x			x
	Culture		[22, 23, 34, 36, 46, 49, 54]	x	x	x		x
	Implementation climate							
		Tension for change	No data					
		Compatibility	[15–18, 20, 21, 23, 25, 27, 28, 31, 33, 36, 37, 40, 41, 43, 46–50, 52, 55–59, 61]	x	x	x		x
		Relative priority	No data					
		Organisational incentives and rewards	[17, 18, 37, 43]	x	x	x		x
		Goals and feedback	[29, 37, 48, 50, 54, 58]	x		x		x
		Learning climate	No data					
		Readiness for implementation	[27, 31–33, 44, 54, 58]	x	x			x
		Leadership engagement	[18, 24, 25, 37, 43, 50, 53, 54, 56, 58]	x		x		x
		Available resources	[17, 18, 25, 28, 31, 34, 37, 39, 41, 45, 49–51, 53]	x	x	x		x
		Access to knowledge and information	[15, 16, 18, 20, 21, 23, 25, 28, 31, 37, 39, 41, 43, 45, 49–58]	x	x	x		x
Characteristics of individuals	Knowledge and beliefs about the intervention		[16, 18, 20–25, 27, 28, 30–34, 37–39, 41, 43–47, 49, 51–54, 56, 57]	x	x	x	x	x
	Self-efficacy		[27]	x				
	Individual stage of change		[31–33, 44]		x			
	Individual identification with organisation		[54, 57]	x				x
	Other personal attributes		[16, 18, 23, 25, 28, 34, 36, 37, 41, 43, 46, 52, 56, 57]	x		x		x
Process	Planning		[16, 18, 24, 27, 31–33, 39–41, 43, 44, 49–51]	x	x	x		x
	Engaging		[21, 31, 37, 50, 54]	x	x			x
		Opinion leaders	[21, 25, 34, 37, 41, 58]	x		x		x
		Formally appointed internal implementation leaders	[18, 27, 32, 39, 44]	x	x			
		Champions	[18, 20, 25, 29, 31, 32, 36, 37, 39, 51, 54, 58]	x	x			x
		External change agents	No data					
		Key stakeholders (Healthcare professional)	[16, 20, 28, 29, 34, 41, 44, 49–51, 53–56, 58]	x	x	x		x
		Innovation participants (patients)	[20]		x			
	Executing		No data					
	Reflecting and evaluating		[16, 24, 29, 31, 34, 41, 43, 48, 50, 51, 53]	x	x	x		x

*MS* management systems, *CS* communication systems, *CD* clinical decision support systems, *IS* information systems, *R* range systems from different e-health domains

Meta-ethnography, as described in depth by Noblit and Hare [13], focusses on interpretation to ‘synthesise understanding’, unlike other approaches to qualitative synthesis, such as thematic analysis, which seek to summarise data [14]. Noblit and Hare describe seven key steps: (i) getting started, (ii) deciding what is relevant to the initial interest, (iii) reading the studies, (iv) determining how the studies are related, (v) translating the studies into one another, (vi) synthesising translations, (vii) and expressing the synthesis. We draw on these steps to consider novel interpretations from combining data within the studies identified. Steps i–iii were incorporated into the steps described above. We provided more detail below in respect of subsequent steps.

### Determining how studies are related

A data extraction form was developed to extract key information and concepts from the included studies and to ease comparison between them. Data were firstly extracted to describe the type of study including publication date, e-health domain, healthcare setting, inclusion and exclusion criteria and methods used. Secondly, the main themes from each review relating to factors that influence implementation of e-health were extracted from both results and discussion sections of the included papers. Data from discussions were included as they often contained further interpretations from the authors, which offered important insights and enhanced the richness of the findings. A summary table created from this matrix with key study details, and summaries of main findings are presented in Additional file 2.

### Translating the studies into one another

Following inductive analysis of the results and discussion sections of the included papers, it became clear that data were, for the most part, consistent with the constructs of the CFIR. Therefore, these detailed data were extracted from the studies into codes guided by the constructs of the CFIR (see Table 2). A category for data that did not fit into one of these constructs or for data that warranted further discussion between reviewers was created. This ensured that data were not being forced into the constructs where there was not a good fit and allowed for the CFIR to be evaluated as to how well the constructs could account for the data from this review. Cells within the matrix remained empty in cases where there were no relevant data in the paper concerned. As a way of remaining faithful to the meanings and concepts of each study, the terminology used in the original paper was preserved within the matrix. A category was also created for the main explanations or theories arising from the authors interpretations that were relevant to the research question. Data were re-categorised from one construct to another and discussions between JR, EM and FS were held until the reviewers were confident that all data were coded into appropriate constructs or categories.

### Synthesising translations

By reading the concepts and interpretations off the matrix, it was possible to establish a sense of the relationships between the studies. It became clear that the relationships between studies seemed to be reciprocal (where concepts of one study encompass another), with many themes occurring across studies which were largely in agreement about the factors that influenced implementation, and from which a line of argument (overarching narrative) could be developed. Following a process of meta-ethnography, meant themes from each of the studies were not just described and compared, but rather interrogated in relation to themes from other studies.

### Expressing the synthesis

There were no data that could not be coded to one of the CFIR constructs, meaning the categories of the CFIR were endorsed and no additional original theoretical insights developed.

## Results

### Search results

A total of 2812 unique citations were identified (see Fig. 1). Of these, 2694 could be excluded on the basis of the title or abstract, leaving 118 citations requiring the full paper before a decision could be made. Of the 118 full papers assessed, 44 [15–58] met the criteria for inclusion and were included in this review (Additional file 2 gives details of all included reviews).

## Description of the included reviews

All studies were published between 2003 and 2014. Fifteen studies originated from Canada [16, 25–29, 31–33, 36, 39, 41, 42, 44, 53], fourteen from the USA [17, 22–24, 30, 35, 38, 40, 49, 52, 54–57], three from the Netherlands [17, 20, 34], three from the UK [37, 46, 51], two from Australia [15, 43] and one each from Germany [47], Sweden [50], Norway [19], Mexico [21], Malaysia [58], Kenya [45] and Israel [48]. All papers were written in English.

Of the 44 studies, 28 focussed specifically on one domain of e-health including: management systems [24, 29] such as electronic medical records [16, 18, 21, 28, 36, 39, 41, 54, 55] or e-prescribing [27]; communication systems such as telemedicine [20, 22, 31, 44, 51], telehealth [17, 32, 33] and home telecare systems [19]; and computerised decision support systems [34, 40, 42, 43, 45, 48] and information systems [38]. Sixteen studies focussed on e-health technologies from across a range of e-health domains [15, 23, 25, 26, 30, 35, 37, 46, 47, 49, 50, 52, 53, 56–58].

Twelve studies were focussed on one particular type of healthcare setting including primary care/ambulatory [15, 24, 27, 28, 36, 39], hospital/inpatient/acute care [22, 29, 40, 46] and home care [19, 53]. Twenty-one studies focussed on the implementation of e-health in to two or more types of healthcare setting [21, 23, 25, 26, 30, 31, 33, 35, 37, 41–45, 47, 49, 50, 52, 54–56]; the remainder of the studies did not define a specific healthcare setting [16, 18, 20, 28, 34, 38, 48, 57, 58].

When judged against the ENTREQ statement [11], many studies were methodologically poor. For example, three [30, 33, 58] did not give details of databases searched and seven searched only one database or source, such as the proceedings of a particular conference [19, 20, 27, 38, 44, 48, 54]. Information about study selection criteria was also inadequate: Sixteen of the 44 studies did not specify the criteria for inclusion or exclusion [16, 17, 20, 22, 30, 32–36, 38–40, 48, 53, 56]. Five did not detail the number of primary studies included in the review [22, 32, 40, 44, 48].

## Factors that influence implementation

The synthesised data from the included studies is presented, classified by the main constructs of the CFIR. There were no data that could not be coded to one of the CFIR constructs. A description of the constructs for which there were most supporting data is described below. A summary of the findings is presented in Table 2 which includes details of the constructs for which there were little or no supporting data and thus not described in the main text. Recommendations for implementation of e-health based on these findings are presented in Table 3.

**Table 3:** Recommendations for implementation based on data from reviews

• Selection of an appropriate e-health system needs careful consideration taking into account:
o Complexity
o Adaptability
o Compatibility with existing systems and work practices
o Cost
• Key stakeholders and implementation champions should be included as early as possible in the implementation process.
• Sufficient financial and legislative support needs to be in place to support implementation.
• Standards for technology which address inter-operability, security and privacy may improve acceptability and implementation.
• Planning implementation is a critical step which includes ensuring that organisations are in a state of readiness.
• The provision of training and education to all those involved with implementation is a key success factor.
• Implementation does not stop with ‘go-live’—there is a need for ongoing monitoring, evaluation and adaptation of systems to ensure intended goals are being met, benefits realised, and ongoing identification of barriers to effective use, along with strategies to overcome these barriers.

## Innovation characteristics

### Adaptability

An important factor in vendor and technology selection, reported by many studies from all e-health domains, was the ability of the technology to be adapted to fit the local context [34, 39]. Technologies that can have technical adjustments made to them to suit the constant modifications of the environment may have greater acceptance and adoption [18, 35, 41, 50, 58]. End user input in the design and development of e-health technologies should be considered as a way of overcoming barriers of adaptability [28].

Related to adaptability is the interoperability of systems reported by many studies [16, 18, 21, 22, 24, 25, 28, 38, 48–50, 52, 53]. To promote their acceptance and use, systems must be able to adequately interface with other IT systems and exchange information [18, 27, 49]. For example, a major barrier to the adoption of Electronic Health Records (EHR) was the inability of new systems to exchange information with systems already in place, due to a lack of consistent data standards [18].

### Complexity

Complexity factors such as slow system performance [55], software and hardware that were difficult to use [27], the need for extensive software modifications [52], the work involved in transferring records between two systems [18], the inability to provide real-time access [38], data handling, reliability, slow speed, unplanned downtime [41] and connectivity issues [49] influence implementation of systems in healthcare settings. Often issues of complexity were linked to health professionals being unable to master the technologies that were implemented [18, 28]. Vendors of e-health systems should aim to make systems as user-friendly as possible, involving end users in the design and development [53], providing guides to their use [34] and providing technical assistance [43].

### Cost

The cost of e-health system and costs associated with their implementation were reported as important implementation factors by the majority of studies across all e-health domains [15–19, 22–25, 27, 28, 30–33, 36, 38, 39, 41, 43, 47, 49, 51–54, 57], with some studies citing cost-related factors as the main barrier to implementation [49, 54]. Cost factors are related to start-up costs, ongoing costs, costs related to a loss of revenue and potential savings to put against these costs.

High set-up costs including purchasing and installation costs were cited as barriers to the initial adoption of e-health systems [49]. Financial incentives to adopt e-health systems from insurers and government agencies facilitated adoption decisions in some case [24, 37, 49].

Concerns about ongoing costs were also reported as barriers to adoption [17, 49]. Evidence of cost-saving and returns on investment were shown to be important in ongoing use of technologies [36, 39]. Establishing cost-effectiveness through formal evaluations, financing of services on a bigger scale, and redesigning business models and incentives were suggested as strategies to help overcome cost-related barriers [20, 24, 51].

## Outer setting

### External policy and incentives

An absence or inadequacy of legislation and policies and liability concerns may hamper the implementation of e-health systems at the organisational and health professional level [17, 18, 20, 51]. The need for recognised standards for the provision of e-health systems was described by many studies [16, 18, 20, 28, 33, 36, 48, 49, 51]. The creation of standards may serve to reduce health professionals’ concerns over patient data safety and professional liability [17, 33] and facilitate the exchange of electronic health information between systems [16] and organisations while maintaining data integrity [49].

Incentives by government organisations and other external stakeholders may facilitate adoption by healthcare organisations [17, 18, 39, 48, 49]. Financial incentives include the provision of initial funds to cover upfront costs [49], financial sponsorship [39], reimbursements for adoption [19, 30, 31, 36], and pay-for-performance initiatives [24, 36, 37, 49].

## Inner setting

### Implementation climate

Implementation climate includes the *compatibility* or general fit between the e-health intervention and the organisation [16, 23, 31, 33, 37, 46, 49, 59]. The fit between e-health systems and workflows in particular was discussed by the majority of studies [15, 16, 18, 21, 23, 25, 27, 28, 36, 40, 41, 43, 47–50, 52, 55–58]. A frequent reason for unsuccessful implementation is that the information systems do not fit well with work practices or daily clinical work [25]. Health professionals’ perceptions that e-health systems disrupt workflows, and the delivery of care, are a barrier to both the implementation and use of these systems [23, 49, 52, 56, 57]. When there is a good fit, or perceived fit, between e-health systems and workflows, and when systems positively influence workplace efficiency, this facilitates use [15, 40, 41]. Incorporating workflow analysis into system design [48, 55], the integration of systems into the usual process of care [15], user-friendly systems [40] and minimising workflow interruptions during implementation [17] may minimise disruptions to workflow.

Alterations to workflows created by the introduction of e-health systems may also disrupt established professional roles, responsibilities [16, 20, 25, 36, 55] and working styles [18]. Physician resistance to e-health implementation is reported by several studies to be related to fear of [18], dissatisfaction with [18] and uncertainty over [43, 47] new roles and responsibilities, created by the introduction of e-health systems [18]. The quality of project management during the implementation period [18], careful study of the downstream effects of implementation on workflow [55], additional training [31, 55], the adaptability of technologies to fit with roles, tasks and workflows [37] and dedicated technical support staff [31] are suggested as strategies to reduce barriers related to disruptions to workflow, roles and responsibilities that e-health implementation may bring.

*Leadership engagement* at all stages of the development and implementation processes can help improve the effective implementation of e-health systems [43, 54, 56, 58] and a lack of involvement can be a barrier to implementation [18, 53]. Management support is also important for implementation success [25, 37, 50, 54].

Authors described *available resources* including the availability of suitable infrastructure as important for implementation success. Infrastructure features included electricity supply [51], available bandwidth [31, 51], access to reliable internet connectivity [45, 51], access to computers [34], electrical power [45] and access to phone lines and mobile phones [45]. The availability, or lack thereof, of time to learn new e-health systems, implement them [25, 37, 53] and train staff to use them [49] was reported by several studies as a resource factor important for implementation. Providing a period of transition in which end users can become familiar with and learn how to use new systems has been advocated [39, 50].

*Access to knowledge and information* was also important for the implementation of systems across all e-health domains. Education was reported to increase staff acceptance of e-health systems [16, 25, 41, 45] including education around anticipated benefits and when those benefits could be expected [54]. A lack of knowledge and a limited understanding of benefits afforded by the systems acted as a barrier to implementation [49, 52, 53]. Nearly all studies made reference to training and support in relation to implementation and acceptance of e-health systems. Generally, access to appropriate, high-quality, well-funded, and easily available training was reported as a facilitator to implementation, whereas it was reported as a barrier when it was non-existent or existent but inadequate [15, 16, 18, 20, 23, 25, 28, 31, 37, 43, 45, 50, 51, 53–58]. Access to ongoing support to use systems was important for system use [21, 25, 28, 31, 37, 39, 43, 54] and a barrier to implementation when it was lacking [21].

## Individual characteristics

### Knowledge and beliefs

Attitudes and beliefs were reported to act as both facilitators and barriers to implementation and acceptance of e-health systems across all e-health domains. Positive attitudes of practitioners toward e-health systems and their implementation increased acceptance and implementation [20, 21, 41, 44, 46, 49], whereas negative attitudes and staff resistance acted as barriers [49, 52]. Positive staff attitudes were described as: beliefs that the new systems would benefit patients [56], interest in the technologies, perceived usefulness and motivation in working with the systems [21]. Negative perceptions included beliefs that electronic systems would disrupt the delivery of care [49]; doubts that these systems can improve patient care, clinical outcomes or improve the quality of medical practices [43]; and distrust in the systems [37] as well as a more general staff resistance to change [28, 32, 39, 41, 49, 51–53]. Strategies to challenge negative attitudes included fostering a culture of communication and cooperation, involving the eventual users of systems in the development and implementation [20, 49], leadership [56], friendly and context-aware user interfaces which promote perceived ease of use and usefulness [21], better education [47], and clearly and prospectively communicating intended benefits and realistic expectations for the system [54]. The attitudes of colleagues [25, 41] and patients [25, 27, 41] were also reported to influence staff attitudes with regard to e-health acceptance as were staff demographic factors [23, 46, 56].

Specifically, fears over a loss of autonomy [16, 18, 27, 34, 37, 39, 43], concerns about liability [16, 18, 22, 37, 43, 51], concerns over patient privacy and security being compromised [16, 18, 20, 23, 24, 27, 28, 30, 31, 33, 39, 41, 43, 45, 47, 49, 51–53, 56, 57], and perceived threats to patient and health professional relationships [18, 25, 27, 28, 31, 34, 37–39, 41, 43, 54, 57] through the introduction of e-health systems were repeatedly reported as barriers to use.

### Other personal attributes

Healthcare professionals’ computer skills, abilities and experience were cited by several studies as influencing implementation and acceptance of e-health systems [16, 18, 25, 28, 34, 36, 37, 41, 43, 52, 57]. Training [25, 37, 43] and financial incentives [37] were cited as strategies to overcome skill-related barriers. Demographic factors such as age, education, sex, nationality, and clinical experience may influence healthcare professionals’ attitudes towards e-health systems [23, 28, 46, 56, 57]; however, most studies conclude that no clear relationships between these characteristics and attitudes could be established [46, 56, 57].

## Process

### Planning

Planning for implementation was important for success, whereas the lack of a strategic plan was reported as a barrier to e-health implementation [24, 27, 31, 44, 50]. The work of planning includes the delineation of roles and responsibilities [43], securing time to invest system selection and procurement [18], evaluating other concomitant policy and process changes [40], needs assessment and analysis, development of a business plan [44], early identification and engagement of champions [31], involving end users [16, 27, 41, 51], establishing a guiding philosophy [33], testing organisational readiness [27, 32], development of incentive and innovation structures [27], communication of the strategy to all staff [50], and development of protocols for using the system and for provision of training [31]. Incremental implementation strategies where features are made available to users according to a plan were cited as preferable to ‘big bang’ approaches to implementation within complex organisations [39, 49]. One review emphasised the need for ongoing effort after the initial ‘go-live’ phase, referring to the ‘under-recognised maintenance phase of implementation’ [29].

### Engaging

The designation of *champions* [18, 20, 25, 29, 31, 32, 36, 37, 39, 51, 54, 58] may be important for implementation success. Engagement of *key stakeholders* in the development and selection of e-health systems and in the planning and execution of implementation processes were important for implementation [16, 20, 28, 29, 34, 41, 44, 49–51, 53–56, 58] through fostering a sense of ownership [20, 44, 51], confidence [28], acceptance [34], enjoyment and self-pride [20] towards the e-health system and increasing buy-in [54].

### Reflecting and evaluating

Evaluation was seen as important to ensure system benefits [29, 48], to increase health professional acceptance through demonstration of benefits [31, 34, 41, 43] and to secure ongoing funding [53], whereas a lack of evaluation and evidence may act as a barrier to implementation [53]. Four reviews included data on the effects of the e-health systems implemented [24, 36, 49, 52]; these reported mixed effects.

### Recommendations

A summary of recommendations for implementation of an e-health system is presented in Table 3.

## Discussion

This review identified and synthesised a large body of literature on the implementation of e-health which covered a wide range of healthcare systems and e-health. Findings suggest that issues around implementation are multi-level and complex. All the included reviews reported multiple factors that were important for implementation, and no single factor was identified as a key barrier or facilitator. The synthesis showed that findings were remarkably consistent across different e-health domains and healthcare settings and well described by the CFIR framework, with no data that did not fit the CFIR.

Key factors for effective implementation included outer context, in particular, the need for supportive legislation, and recognised standards. The fit of e-health systems with current organisational workflow was another key factor.

In comparing the findings of this review with the one it updates [7], it appears that many implementation factors are consistent over time, such as the prevailing focus on organisational issues including the need for adequate resources, particularly financial, policy support, standards and interoperability. This suggests that although e-health may be a rapidly changing field, many of the challenges of implementing systems within organisations remain constant over time. However, some notable differences exist between the findings of the two reviews. The original review reported a concentration on organisational issues within the literature. Although also strongly present in this review, the use of the CFIR sensitised the focus of analysis to other factors as well including factors related to the innovation, outer context, individuals and the process of implementation. The original authors reported that very little attention had been paid in ensuring that the potential benefits of new technologies are made transparent through ongoing evaluation and feedback. In this review, there was a focus given to the role of reflecting and evaluating which may represent an increased awareness of their importance in implementation.

The results from this review are comparable to another large systematic review of reviews by Lau et al. which synthesised the literature on the barriers and facilitators to the implementation of complex innovations within primary care settings [60]. Both reviews highlighted the importance of policies and incentives; adequate infrastructure and resources; engagement of key personnel; organisational readiness; individuals’ knowledge and beliefs; and the fit of innovations with workflows, processes and systems. As such, it seems these factors are important for implementation across interventions and healthcare settings. Lau et al.’s review found that the perceived benefits or harm of implementation such as expectations of more efficient workflow or lower productivity were only an important factor for the implementation of e-health interventions and was not present in the data for other types of interventions (guidelines or evidence-based practice, management of care, public health or preventative medicine, integration of new role or collaborative working). Adaptability and cost were only present in the data for e-health interventions and one other type of intervention. These factors were given a lot of focus in the current review thus suggesting that these factors may be unique or particularly relevant to e-health implementation.

## Methodological strengths and weaknesses

Conducting a systematic review of reviews, given the enormous literature reporting on the implementation of e-health, provided a useful and economical way to manage evidence across a broad topic area. This review allowed the findings of many separate reviews to be compared and contrasted and provided a summary of evidence from reviews which focussed on different e-health interventions and different healthcare settings. The use of a meta-ethnographic approach provided a rigorous and transparent approach to the data analysis and the translation of the studies into one another, which allowed the development of an overarching narrative which endorsed the value of the CFIR framework.

Although this review was rigorous, carefully executed and employed a robust methodological approach, it has several limitations. Systematic reviews, and also the studies included in them, may be subject to publication bias. Reviews of reviews inevitably result in a time-lag, as new primary data must first be published, then included in a review and then into a review of reviews. Other limitations include the fact that this review was dependent on the interpretations of primary data provided by the authors of included reviews. It was often not clear whether the data came from the primary studies or were subsequent interpretations by the authors of included reviews. Many reviews did not specify whether the data came from clinicians, nurses, other primary care staff or multidisciplinary teams; therefore, it was not possible to differentiate the perspectives of specific roles (e.g. nurses). We recommend that authors of implementation studies adhere to reporting guidelines, such as the forthcoming Standards for Reporting Implementation Studies (StaRI) (Pinnock et al., submitted for publication). Finally, the data available for the synthesis was limited by what previous reviewers included in their reviews, and therefore, it is not possible to tell whether the areas of the CFIR which are not addressed are not important or just not addressed.

## Conclusions

We took a multi-level approach to synthesise data from 44 reviews, addressing factors important for the implementation of e-health across healthcare settings. The use of the CFIR highlighted that the individual e-health technology, the outer setting, the inner setting, the individual health professionals, and the process of implementation are all important for implementation and should be considered carefully when attempts are made to implement e-health into health systems. Particular consideration should be paid to the fit of e-health with external and internal contexts. The use of the CFIR allowed the identification of areas that received little attention in the literature which may represent potential themes for future research including the *source* and *trialability* of e-health systems, the *relative priority* given to the systems, the role of *external change agents* and the involvement of *innovation participants* (patients).

## Additional files

Additional file 1: MEDLINE search strategy. (DOCX 15 kb)
Additional file 2: Summary details of the 44 included studies. (DOCX 30 kb)

## Abbreviations

CFIR: Consolidated Framework for Implementation Research
ENTREQ: Enhancing transparency in reporting the synthesis of qualitative research
NHS: National Health Service
PRISMA: Preferred Reporting Items for Systematic Reviews and Meta-Analyses
