# Supplementary material for: Factors that influence the implementation of e-health: a systematic review of systematic reviews (an update)
Source: Implement Sci. 2016 Oct 26;11:146. doi: 10.1186/s13012-016-0510-7 (PMC5080780; doi:10.1186/s13012-016-0510-7)
Supplement: Additional file 1: — MEDLINE search strategy. (DOCX 15 kb) [file 13012_2016_510_MOESM1_ESM.docx]

# Additional File 1: MEDLINE search strategy

1. exp Medical Informatics Applications/
2. exp Management Information Systems/
3. exp Decision Making, Computer-Assisted/
4. exp Diagnosis, Computer-Assisted/
5. exp Therapy, Computer-Assisted/
6. exp Medical Records Systems, Computerized/
7. exp Medical Order Entry Systems/
8. exp Electronic Mail/
9. exp Videoconferencing/
10. exp Telemedicine/
11. exp Computer Communication Networks/
12. exp Internet/
13. 1 or 2 or 3 or 4 or 5 or 6 or 7 or 8 or 9 or 10 or 11 or 12
14. "Routin*"[title, original title, abstract, name of substance word, subject heading word]
15. “Normali?*”[title, original title, abstract, name of substance word, subject heading word]
16. "Integrat*"[title, original title, abstract, name of substance word, subject heading word]
17. "Facilitate*"[title, original title, abstract, name of substance word, subject heading word]
18. "Barrier*"[title, original title, abstract, name of substance word, subject heading word]
19. "Implement*"[title, original title, abstract, name of substance word, subject heading word]
20. "Adopt*"[title, original title, abstract, name of substance word, subject heading word]
21. 14 or 15 or 16 or 17 or 18 or 19 or 20
22. 13 and 21
23. limit 22 to (meta analysis or systematic reviews)
24. limit 23 to yr="2009 -Current"
