# Supplementary material for: Factors that influence the implementation of e-health: a systematic review of systematic reviews (an update)
Source: Implement Sci. 2016 Oct 26;11:146. doi: 10.1186/s13012-016-0510-7 (PMC5080780; doi:10.1186/s13012-016-0510-7)
Supplement: Additional file 2: — Summary details of the 44 included studies. (DOCX 30 kb) [file 13012_2016_510_MOESM2_ESM.docx]

Additional File 2: Summary details of the forty-four included studies

| Author | Date (date published online if different) | Country of origin of 1st author | Aim/purpose | Setting | e-health domain | Number of papers included | Summary of main factors that influence e-health implementation |
| --- | --- | --- | --- | --- | --- | --- | --- |
| Adaji *et al.* | 2008 | Australia | To demonstrate the benefits of information technology in supporting a systematic approach to diabetes management in general practice and to increase understanding of perceived barriers to and facilitators to the use of information technology in this context. | Primary care | Range of e-health domains | 29 | Barriers: confidentiality concerns, inadequate funding, workforce shortages, lack of time and anxiety about change. Facilitators: Adequate training and integration into the usual process of care. |
| Archer *et al.* | 2011 | Canada | To review the literature on personal health record systems and to describe the design, functionality, implementations, applications, outcomes, and perceived and real benefits of personal health record systems, with an emphasis on experience in the USA and Canada. | Not defined | Management systems | 130 | PHR adoption is dependent on growth in electronic medical record adoption. Patient-oriented functionalities need to be provided to support self-management and disease prevention. |
| Benavides-Vaello *et al.* | 2013 | USA | To explore the advances and uses of telecommunications technology, and related issues, in the delivery of mental health and substance abuse treatment services within rural areas. | Mental health and substance abuse treatment services within rural areas | Communication systems | 38 | Costs; financial sustainability was one of the primary barriers to expansion of telehealth services in rural areas. |
| Boonstra and Broekhuis | 2010 | Netherlands | To identify, categorise, and analyse barriers perceived by physicians to the adoption of electronic medical records in order to provide implementers with beneficial intervention options. | Not defined | Management systems | 22 | Financial, technical, time, psychological, social, legal, organisational and change process. |
| Botsis *et al.* | 2008 | Norway | To review the literature on home telecare for elderly patients suffering from chronic diseases | Homecare | Communication systems | 54 | Organisational, ethical, legal, design and usability issues need to be addressed before widespread implementation |
| Broens *et al.* | 2007 | Netherlands | To identify the determinants that influence the implementation of telemedicine initiatives. | Not defined | Communication systems | 45 | Factors categorised as: technology, acceptance, financing, organization and policy and legislation |
| Castillo *et al.* | 2010 | Mexico | To identify the critical adoption factors for electronic health records by physicians and to use them as a guide to support their adoption process automatically. | Mixed | Management systems | 68 | User attitude towards information systems, workflow impact, interoperability, technical support, communication among users, and expert support |
| Demaerschalk *et al.* | 2009 | USA | To describe the technology that makes stroke telemedicine possible, the members that should be included in a telestroke team, the hub-and-spoke characteristics of a telestroke network, and the format of a typical consultation. | Hospitals | Communication systems | not stated | The long-term sustainability and growth of telestroke practice remain threatened by unresolved legal, economic, and market factors |
| Fitzpatrick *et al.* | 2008 | USA | To gain a better understanding of communication problems in healthcare settings and identify gaps in the research. | Mixed | Range of e-health domains | 98 | Social and organisational culture |
| Fontaine *et al.* | 2010 | USA | To gain a fuller understanding of the factors associated with health information exchange adoption by primary care practices. | Primary Care | Management systems | 64 | Cost, security and privacy issues, liability, leadership, strategic planning, competition, and technical barriers |
| Gagnon *et al.* | 2012 (2010) | Canada | To systematically review factors that are positively or negatively associated with information and communication technology adoption by healthcare professionals in clinical settings. | Mixed (mostly hospitals) | Range of e-health domains | 101 | Facilitators: Perception of the benefits of the innovation and ease of use. Limiting factors: design, technical concerns, familiarity with ICT, and time. |
| Gagnon *et al.* | 2009 | Canada | To carry out a systematic review of the effectiveness of interventions to promote the adoption of information and communication technologies by healthcare professionals. | Mixed | Range of e-health domains | 10 | Training and audit and feedback. |
| Gagnon *et al.* | 2014 (2013) | Canada | To review users’ perceptions of barriers and facilitators to e-prescribing and implementation in primary care. | Primary care | Management systems | 34 papers accounting for 28 studies | Technical and organisational support. |
| Goldstein *et al.* | 2014 (2013) | Canada | To examine physician barriers to adopting electronic medical records as well as anaesthesiologists’ experiences. | Not defined | Management systems | 14 papers accounting for 12 studies | Identified barriers to adoption of were financial, technological, and time constraints. |
| Gruber *et al.* | 2009 | Canada | To determine the current evidence about the process and outcomes of the implementation of clinical information systems in healthcare facilities. | Acute care facilities | Management systems | 18 | Clinical context and end user support |
| Hsieh and Lin | 2011 | USA | To identify benefits and challenges of the development and implementation of electronic medical records, tele-health, and electronic appointment reminders. | Mixed (primary care and behavioural healthcare/mental health settings) | Range of e-health domains | 42 | Personnel resistance and fear; availability of training, support and other resources for personnel; security and confidentiality; and infrastructure development to enhance clinical workflow, billing and collection activities, and quality assurance measures. |
| Jarvis-Selinger *et al.* | 2008 | Canada | To provide policymakers, administrators, and healthcare professionals with an evidence-based foundation for informed decision-making regarding videoconferencing | Mixed | Communication systems | 225 | Necessary technical conditions need to be in place and key strategies for organisational readiness and technology adoption |
| Jennett and Brandstadt | 2005 | Canada | To detail and critique the published international peer-reviewed studies that have focused on assessing telehealth readiness for rural and remote health. | Rural | Communication systems | not stated | An appreciation of practice context, strong leadership, and a perceived need to improve practice. |
| Jennett *et al.* | 2004 | Canada | To inform future telehealth policy directions regarding the socioeconomic impact of telehealth | Mixed | Communication systems | 57 | Barriers and facilitators included confidentiality, reimbursement, and legal and ethical considerations. To become fully integrated into the health care system, telehealth must be viewed as more than an add-on service. |
| Kilsdonk *et al.* | 2011 | Netherlands | To systematically review the status quo on what is known on factors contributing to clinical decision support systems acceptance. | Not defined | Clinical Decision Support Systems | 29 | Human (system use and user satisfaction), organization (structure and environment and technology (system quality, information quality and service quality |
| Kukafka *et al.* | 2003 | USA | A systematic literature analysis was conducted to confirm the assertion that the literature on information technology use behaviour does not include a multi-level approach | Mixed (academic non-medical, government agency, academic medical centres/hospitals, businesses and other organization) | Range of e-health domains | 24 | Preparing the environment for change and providing economic resources, incentives, and social support to facilitate the change. A comfortable and flexible environment. User knowledge of, and belief in, the new system. Functional system which are compatible with the target population. |
| Lau *et al.* | 2012 | Canada | To examine the impact of electronic medical records in the physician office, factors that influenced their success and the lessons learned. | Ambulatory | Management systems | 43 | Micro-level: technical design, performance and support affected usage and user satisfaction. Meso-level: the implementation process and resulting workflow. Macro-level: incentives such as pay-for-performance were seen as an important driver for adoption. |
| Lluch | 2011 | UK | To identify the barriers to health information technology adoption from an organisational management perspective | Mixed | Range of e-health domains | 79 | Structure of healthcare organisations, Tasks, People policies, Incentives; and Information and decision processes |
| Lu *et al.* | 2005 | USA | To examine the potential benefits of personal digital assistants as factors that will promote their adoption and identify barriers to their acceptance in health care. | Any | Range of e-health domains | 95 | Major barriers to adoption were identified as usability, security concerns, and lack of technical and organisational support |
| Ludwick and Doucette | 2009 | Canada | To identify the current state of knowledge about health information systems adoption in primary care and understand factors affecting implementation outcomes. | Primary care | Management systems | 86 | System design, project management, procurement and users’ previous experience effected implementation |
| Mack *et al.* | 2009 | USA | To review the use of clinical decision support systems available in the paediatric intensive care unit. | Paediatric intensive care unit | Clinical Decision Support Systems | not stated | Workflow, technological issues and change management |
| McGinn *et al.* | 2011 | Canada | To categorize, synthesize, and compare the perspectives of targeted groups of users (public, patients, health care professionals and managers) and to underline factors influencing electronic health record implementation specific to each user group. | Mixed | Management systems | 60 | Design and technical concerns, ease of use, interoperability, privacy and security, costs, productivity, familiarity and ability with EHR, motivation to use EHR, patient and health professional interaction, and lack of time and workload. |
| Mollon *et al.* | 2009 | Canada | To evaluate whether certain features of prescribing decision support systems predict successful implementation, change in provider behaviour, and change in patient outcomes. | Mixed (hospital and ambulatory) | Clinical Decision Support Systems | 41 | A lack of attention to evidence-based optimization  of CDSS interventions hampers  the development and implementation |
| Moxey *et al.* | 2010 | Australia | To explore the barriers to, and facilitators of, clinical decision support systems uptake by physicians to guide prescribing decisions. | Mixed (ambulatory and inpatient) | Clinical Decision Support Systems | 60 papers accounting for 58 studies | Availability of hardware, technical support and training; integration of the system into workflows; and the relevance and timeliness of the clinical messages |
| Ohinmaa | 2006 | Canada | To assess telemedicine projects outside the USA and provide examples of promising results that could be disseminated to other countries. | Mixed (non USA) | Communication systems | not stated | Success factors: key persons’ involvement in planning and implementation, extensive planning, the attitudes of participants, remote location, planning readiness, leadership readiness, workplace readiness and technical readiness Factors contributing to failure: inadequate needs assessment, lack of ‘buy-in’, lack of staff preparation and resistance to change. |
| Oluoch *et al.* | 2012 | Kenya | To identify studies on electronic medical record based clinical decision support systems describing process and outcome measures and reported barriers to implementation. | Mixed (inpatient and outpatient settings) | Clinical Decision Support Systems | 12 | Technical infrastructure problems |
| Oroviogoicoechea *et al.* | 2008 | UK | To review existing nursing research on inpatient hospitals’ information technology systems in order to explore new approaches for evaluation research on nursing informatics to guide further design and implementation of effective information technology systems. | Inpatient hospital setting | Range of e-health domains | 39 | Social and organisational contexts |
| Orwat *et al.* | 2008 | Germany | A systematic overview and analysis of system developments and implementations of pervasive computing in healthcare and highlighting experiences in deployment. | Mixed (ambulatory, home and mobile, clinical, care and rehabilitation) | Range of e-health domains | 69 (describing 67 studies) | Organisation, personnel, privacy concerns and financial issues. |
| Peleg | 2006 | Israel | To understand the challenges facing developers of clinical decision support systems. | Not defined | Clinical Decision Support Systems | not stated | The goals of CDSS are important in developing successful, usable clinical decision support systems as it is the vision that drives the way they are developed, implemented, integrated with the environment, and evaluated. |
| Police *et al.* | 2010 | USA | To better understand current utilisation rates along with benefits and barriers to health information technology adoption in physician practice organisations. | Mixed (physician practice organisations) | Range of e-health domains | 119 | The largest barrier to HIT adoption in physician groups is the high initial and ongoing costs of electronic systems. Lack of sufficient training, a disorganised or non-receptive practice culture and technological problems such as inadequate connectivity appear to impede effective HIT use. |
| Rahimi *et al.* | 2009 | Sweden | To organize the knowledge gained in qualitative studies performed in association with healthcare information systems implementations and to use this knowledge to outline an updated structure for implementation planning | Mixed (primary care and hospitals) | Range of e-health domains | 17 | Education and training support, information needs assessment, implementation process, management support, work routines and workflow, motivation and rationales, integration of the system, trust, technical system performance, participation and user involvement, system effectiveness. |
| Saliba *et al.* | 2012 | UK | To systematically identify factors that hinder or support implementation of cross-border telemedicine services. | Cross-border telemedicine services | Communication systems | 94 | Legal factors; sustainability factors; cultural factors; and contextual factors. |
| Shekelle *et al.* | 2006 | USA | To assess the evidence base regarding benefits and costs of health information technology systems. | Mixed (ambulatory, inpatient and outpatient) | Range of e-health domains | 256 of which 20 focussed on barriers and facilitators to implementation | Barriers were classified as; situational barriers (including time and financial concerns), cognitive and or physical barriers (include physical disabilities and insufficient computer skills), liability barriers (including confidentiality concerns), and knowledge and attitudinal barriers. |
| Stolee *et al.* | 2010 | Canada | To identify barriers, facilitators, and recommendations for using electronic health information systems in home care settings. | Homecare | Range of e-health domains | 45 | Costs, especially during implementation, training, and lack of user acceptance/staff resistance. The most common facilitators included portable technology, strategies to decrease data entry errors, and managerial support and user incentives |
| Studer | 2005 | USA | To systematically review studies assessing the effect of organisational factors on the effectiveness of electronic medical records system implementation. | Mixed (physician practices, group medical practices, hospitals and academic health centres) | Management systems | 23 | Factors influencing the effectiveness of EMR system implementation included management support, financial resource availability, implementation climate and implementation policies and practices. |
| Vreeman *et al.* | 2006 | USA | To identify, review, and summarize the benefits, barriers, and key factors for success in implementing electronic health records in physical therapist practice settings. | Mixed (physical therapy settings) | Management systems | 13 | Essential conditions for successful implementation: workflow analysis, involvement of end users, significant resources for training, adequate software and hardware performance, commit to data standards. |
| Waneka and Spetz | 2010 | USA | To determine the impact of health information technologies on nurses and nursing care. | Mixed (hospital and ambulatory) | Range of e-health domains | 74 | Effective leadership and involvement from HIT users at all stages of the development and implementation processes can help improve the effective implementation |
| Yarbrough and Smith | 2007 | USA | To increase understanding of physician technology acceptance and barriers to such acceptance, | Any | Range of e-health domains | 18 | Time/practice-related issues, organisational issues, personal issues, and system-specific characteristics influence a physician’s acceptance of a new technology. |
| Yusof *et al.* | 2007 | Malaysia | To present the main findings of a systematic review of selected case studies on healthcare information systems adoption in clinical practices. | Not defined | Range of e-health domains | 55 | Critical adoption factors: technology (ease of use, system usefulness, system flexibility, time efficiency, information accessibility and relevancy); human (user training, user perception, user roles, user skills, clarity of system purpose, user involvement); organisation (leadership and support, clinical process, user involvement, internal communication) as well as the fit between them. |
